# Safety and efficacy of G2-S16 dendrimer as microbicide in healthy human vaginal tissue explants

**DOI:** 10.1186/s12951-022-01350-8

**Published:** 2022-03-21

**Authors:** I. Rodríguez-Izquierdo, M. J. Serramía, R. Gómez, G. Espinosa, M. Genebat, M. Leal, M. A. Muñoz-Fernandez

**Affiliations:** 1 Head Immunology Section, Laboratorio InmunoBiología Molecular, Hospital General Universitario Gregorio Marañón (HGUGM), 28007 Madrid, Spain; 2 Spanish HIV-HGM BioBank, 28007 Madrid, Spain; 3 Instituto de Investigación Sanitaria Gregorio Marañón (IiSGM), 28007 Madrid, Spain; 4 Departamento de Química Orgánica E Inorgánica, Instituto de Investigación Química “Andrés M. del Río” (IQAR), Universidad de Alcalá (UAH), 28805 Alcalá de Henares, Spain; 5 Networking Research Center On Bioengineering, Biomaterials and Nanomedicine CIBER-BBN, Madrid, Spain; 6 Centro de Ginecología Y Diagnóstico Prenatal “Dr. Lorenzo Chacón”, Hospital Viamed Santa Ángela de La Cruz, Sevilla, Spain; 7 Servicio de Medicina Interna, Hospital Fátima, Sevilla, Spain; 8 Servicio de Urgencias, Hospital Universitario Virgen del Rocio, Sevilla, Spain; 9 Unidad de Inmunovirología, Servicio Medicina Interna, Hospital Viamed Santa Angela de La Cruz, Sevilla, Spain

**Keywords:** Vaginal explants, G2-S16, HIV-1, Microbicide, Safety, Efficacy

## Abstract

**Background:**

The absence of an effective treatment and vaccine in HIV-1 pandemic place preventive strategies such as safety and effective microbicide development as a central therapeutic approach to control HIV-1 pandemic nowadays.

**Results:**

Studies of cytotoxicity, immune population status, inflammation or tissue damage and mainly prophylactic inhibition of HIV-1 infection in vaginal human explants demonstrate the biosafety and effectivity of G2-S16 dendrimer. Human explants treated with G2-S16 dendrimer or treated and HIV-1 infected do not presented signs of irritation, inflammation, immune activation or T cell populations deregulation.

**Conclusions:**

Herein we conclude that G2-S16 dendrimer has demonstrated sufficient efficacy, biosafety, effectivity and behavior in the closest to the real-life condition model represented by the human healthy donor vaginal tissue explants, to raise G2-S16 dendrimer as a promising candidate to clinical trials to develop an effective microbicide against HIV-1 infection.

**Graphical Abstract:**

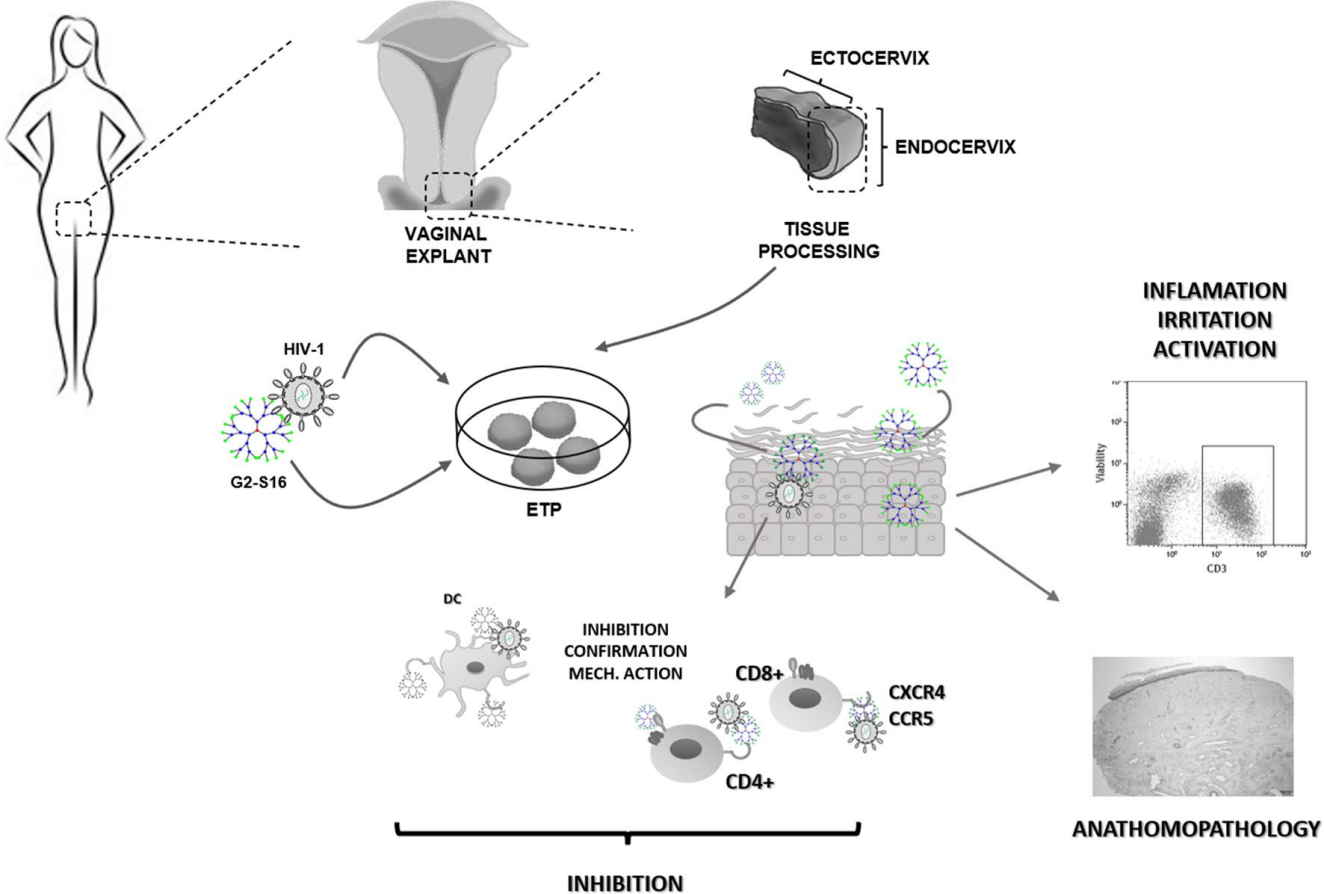

## Background

Sexual transmitted infections (STI) are one of the most prevalent diseases globally. It is estimated that more than a million of STI cases occur every day [1]. Sexual infections are mainly caused by bacterial or viral pathogens. In this sense, UNAIDS estimated that 37 million people are infected by HIV-1 around the world and 2 million of new infections are detected every year [2]. Moreover, other viral pathogens such as human papilloma virus or herpes simplex virus have a huge prevalence in the population. More than 290 million women are infected by human papillomavirus (HPV), one of the most common STI, and more than 530 million people are infected with the herpes simplex virus-2 (HSV-2) [3, 4].

Focusing on HIV-1 or HSV-2 viral infections, current treatments are only focused on mitigating or modulating pain and treating clinical symptoms, but not on preventing infections or on curing them. Despite all efforts made, to achieve an effective cure against HIV-1 infection is still an elusive goal. The only one effective preventive treatment to date is the pre-exposure prophylaxis (PreP) [5]. However, PreP is not available to a widespread risky population due to the limited availability and excessive cost. Another important factor related to PreP effectivity is related to the timeline treatment. PreP must be administered a week before viral exposure to have optimum efficacy [6–8]. Therefore, adherence still remains one of the major problems related to prophylaxis. In addition, PreP being a combination of antiretroviral (ARV) (tenofovir and emtricitabine) favors side effects and resistance appearance [9, 10].

Related to treatments, nanotechnology has emerged as a viable therapeutic alternative to develop new drugs and treatments against diverse pathogens. In the case of viral infections, we have previously described the potent efficacy of dendrimers against viruses such as HIV-1 and HSV-2, in vitro as well as in vivo [11–15]. Dendrimers are hyperbranched nanoparticles consisting of a central core and several functional groups in the periphery. Depending of the dendrimer construction, they could be capable of encapsulating several drugs inside the scaffolding of the dendrimer [16, 17]. Those characteristics pose dendrimers as enormous versatile particles capable of acting against almost any phase of the viral cycle. Concretely, G2-S16 dendrimer, a sulfonate anionic carbosilane dendrimer, demonstrated to be safe in mice and rabbits, impeding the infection by HIV-1 in an 80% in humanized mice [18, 19].

A massive number of clinical trials have been developed against HIV-1 infection to achieve new treatments and therapeutic, preventive vaccines, however most of them have failed [20]. The main reason of failure is related to the lack of drug efficacy and the unexpected toxicity, once treatments were extrapolated to humans [21, 22]. Those variables caused by preclinical assays used for ARV or drugs developments are not accurate enough. It happens either because of animal models or because oversimplified conditions have been used in vitro or because of different physiological environments, not similar to those of humans [23].

While it is true that physio-pathology of the human vaginal tract is not presented in the animal models mentioned (mice and rabbits), results obtained with G2-S16 dendrimer indicate a similar behavior when extrapolated to clinical trials. On the other hand, it is important to note that a huge number of nanoparticles presents a great tolerance and inhibition in vitro and in vivo, however, at the point of the extrapolation to human, episodes of inflammation, irritation or physiology alterations appear [24, 25]. Considering this, it would be crucial to test the behavior of G2-S16 dendrimer in a human vaginal environment to reach clinical trials. In that sense, the main objective would be to demonstrate the efficacy against HIV-1 infection and the safety of G2-S16 dendrimers in healthy human vaginal explants.

## Results

### Tissue viability and biocompatibility of G2-S16 dendrimer

**Fig. 1:**
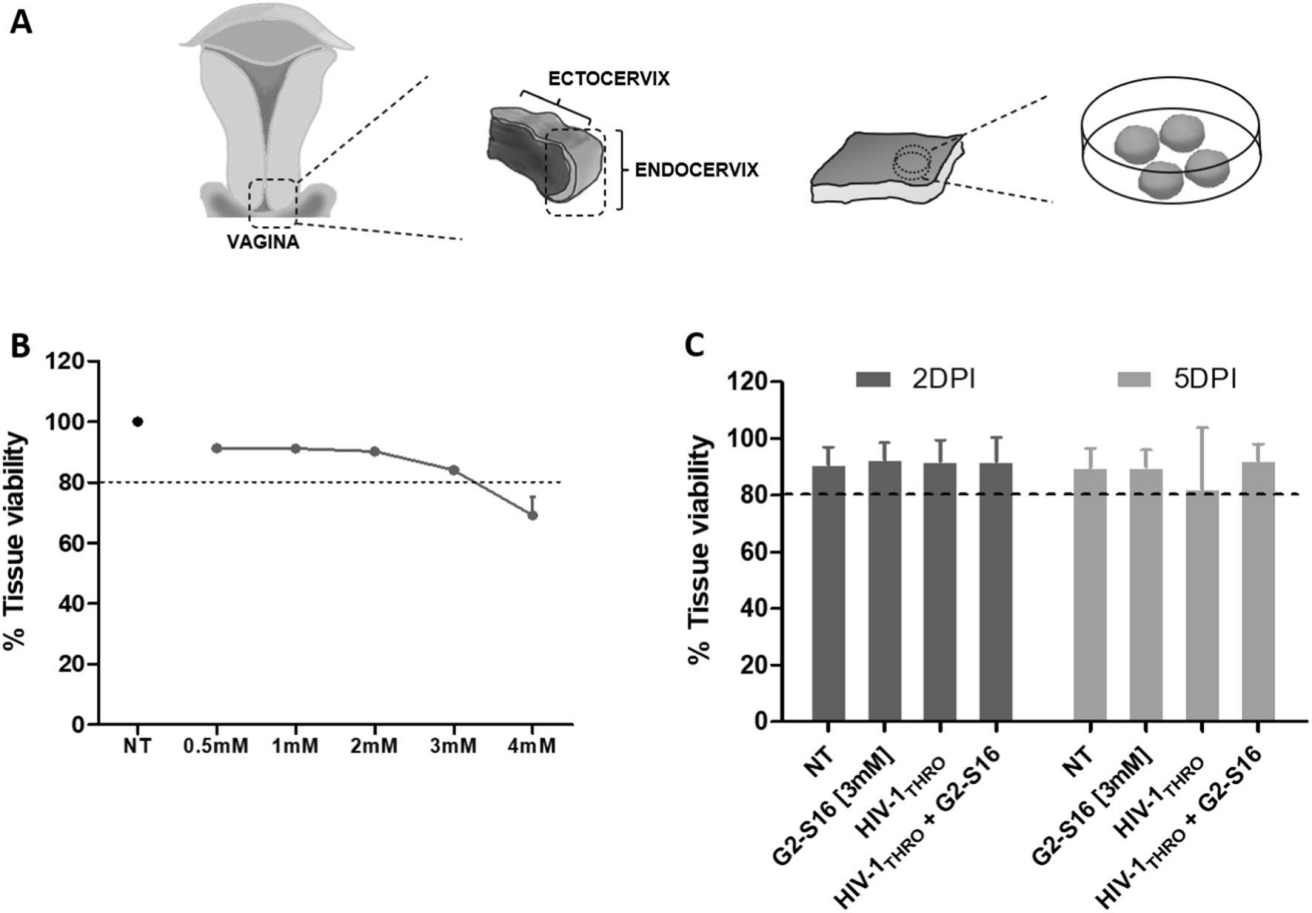
Biocompatibility of G2-S16 dendrimer.** A** Schematic representation of ETP processing. **B** Biocompatibility of G2-S16 dendrimer at 5 days. **C** Tissue viability after 48 h or 5 days post-infection. Data represent mean of n = 3 (**B**) and n = 5 (**C**) different ectocervix tissue explants made by duplicate (4 ETP/conditions). Graphics **B** and **C** represent tissue viability ± SD. *NT* Non-treated ETP, *DPI* days post-infection

Tissue explants were received within 24 h after surgery and processed as mentioned in “Material and methods” section (Fig. 1A). The first step was to determine the tissue viability to assess the possible damage due to the transport and to the surgery itself. All tissue explants were first flow cytometry viability tested by aqua^®^ (*data not shown*) and only optimal tissues were next processed for in ectocervix tissue portions (ETP) ex vivo assays.

Portions obtained for ectocervix sections were further tested for G2-S16 dendrimer toxicity. ETPs were treated in a concentration range (0.5–4 mM) to determine the biocompatibility of G2-S16 dendrimer after 48 h or 5 days of incubation (Fig. 1B). Data obtained set the maximum non-toxic concentration for G2-S16 dendrimer at 3 mM for further assays.

Once G2-S16 dendrimer biocompatibility was deduced, ETPs were treated with G2-S16 dendrimer (3 mM), infected with HIV-1_THRO_ isolate (7500 TCID_50_) or treated and infected for 2 or 5 days, and analyzed for tissue viability (Fig. 1C) in order to determine that the possible effects on HIV-1 inhibition or behavior in tissue is due to the direct effect of G2-S16 dendrimer and not due to the damage produced by HIV-1 or G2-S16 dendrimer treatments of ETP. Our data demonstrated that tissue viability is not compromised by the different infections or treatments, and all conditions tested in this assay are viable enough.

### HIV-1 replication in human ETP explants

Cervicovaginal tissue is susceptible to be infected by soaking ETP in a viral suspension. As deduced in the viability assay, G2-S16 dendrimer 3 mM was tested for HIV-1 inhibition in cervicovaginal explants. The ETPs were infected with HIV-1_THRO_ T/F isolate (7500 TCID50), treated with G2-S16 dendrimer (3 mM) or pre-treated and infected. Supernatants were collected at 2- or 5-days post-infection (DPI) and titrated on TZM.bl HIV-1 reporter cell line. Additionally, ETPs were tested for viability assay to ensure that tissues are viable and the possible inhibition is due directly to the G2-S16 dendrimer effect and not to the tissue death that could reduce luminescence signal.

**Fig. 2:**
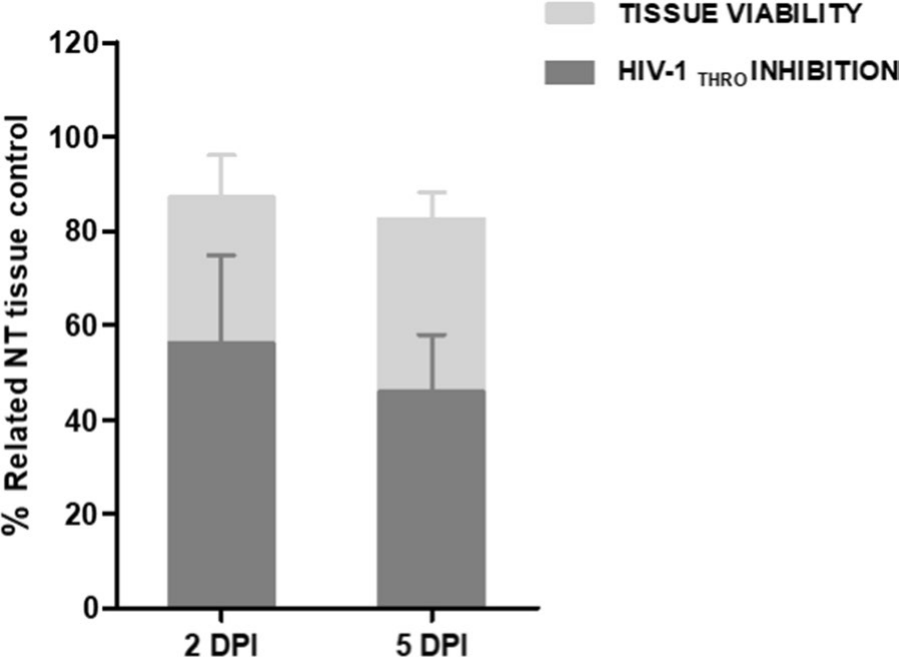
G2-S16 dendrimer inhibition of HIV-1 infection. ETP of ectocervix explants were infected with HIV-1 THRO T/F isolate or treated with G2-S16 dendrimer 3 mM and infected. Tissue viability measured by MTT assay and HIV-1 inhibition titrated on TZM.bl reporter cell line after 2 or 5 DPI were represented. Bars represent the mean of 5 different ectocervix tissue explants made by duplicate (4 ETP/condition) ± SD. *NT* non-treated, *DPI* days post-infection

Data showed a significant inhibition at 2 DPI (56% inhibition) as well as 5 DPI (46% inhibition) (Fig. 2). Viability of ETP also reinforces inhibition data that inhibition is only due to direct action of G2-S16 dendrimer (Fig. 2).

**Fig. 3:**
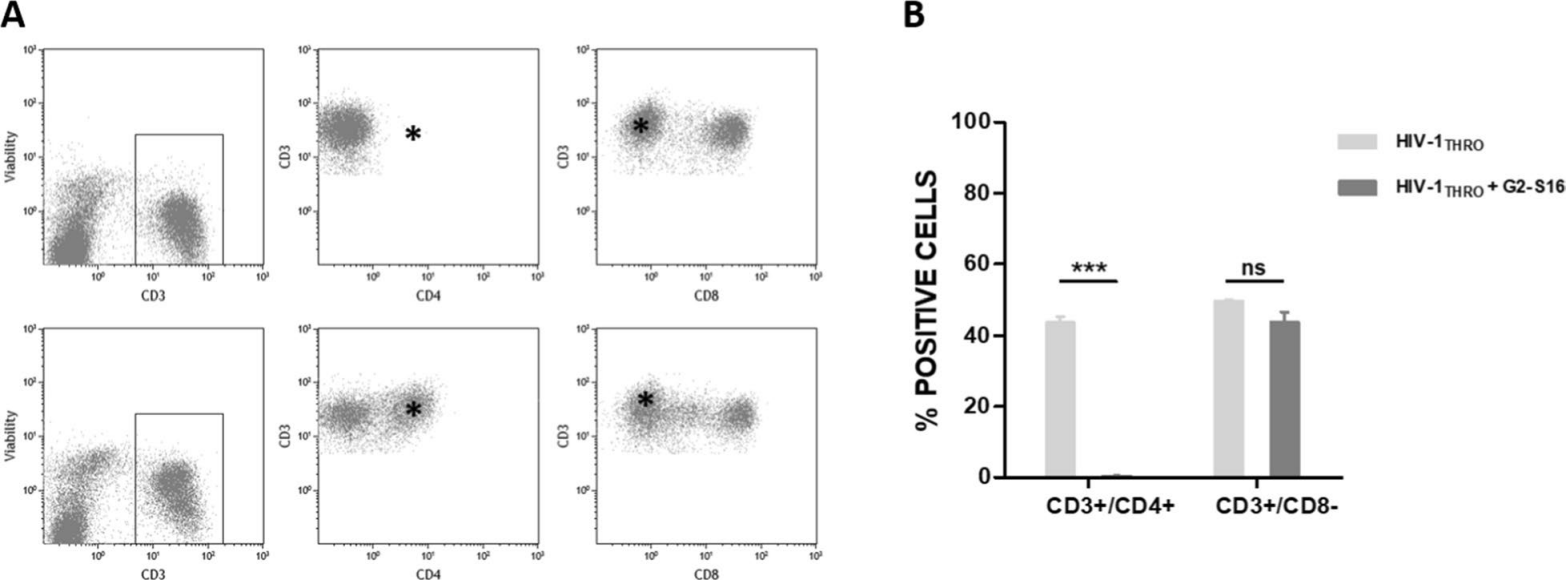
Lymphocyte relative abundance in tissue explants. **A** Representative flow cytometry analysis of CD4 and CD8 abundance in ETP after HIV-1 infection and G2-S16 dendrimer 3 mM treatment (upper panels) or HIV-1 infection solely (lower panels) **B** Analysis of ectocervix explant T cell population relative abundance (n = 5 ectocervix explants) performed on 4 ETP/condition

Additionally, ETPs were digested with collagenase IV, and stained to set the immune status of lymphocyte CD4^+^ and CD8^+^ population as well to corroborate viability by flow cytometry analysis. Interestingly, ETP which were treated with G2-S16 dendrimer loose CD4 staining (Fig. 3A). This fact caused by G2-S16 dendrimer has shown that CD4 receptors bind in host cells and destabilize the GP120-CD4 complex, blocking the HIV‑1 entry into CD4^+^ T cells.

When ETP were HIV-1 infected, CD4 population remained unalterable (no differences between CD3^+^CD4^+^ and CD3^+^CD8^−^) (Fig. 3A lower panels and Fig. 3B). However, in ETP treated with G2-S16 dendrimer and infected, the CD4 population disappears (Fig. 3 asterisk, upper panels) even if CD3^+^ CD8^−^ demonstrate that it is not due to a real loss of CD4 population (Fig. 3B).

### Effect of HIV-1 and G2-S16 dendrimer treatment in T-cell activation

Once confirmed that G2-S16 dendrimer inhibits HIV-1 infection ex vivo, the next step was to assess whether the effect of dendrimer in ectocervix explants generates an activation or deregulation on T cell population.

To test this hypothesis, ETPs were treated with G2-S16 dendrimer 3 mM and compared with non-treated. ETPs were digested and disaggregated, and resulting cells were immune-stained for activation HLA-DR^+^, CD38^+^ or double positive HLA-DR^+^CD38^+^ markers on T CD8^+^ cells as well as T CD4^+^ cells. Flow cytometry analysis shows that treatment with G2-S16 dendrimer does not affect activation parameters either on CD8^+^ or CD4^+^ populations (Table 1).

**Table 1:** Effect of G2-S16 dendrimer treatment in T cell ectocervix explants activation

T-Cell activation	Coefficient	P
T CD3^+^CD4^+^		
HLA-DR^+^	− 0.818	0.371
CD38^+^	− 0.635	0.263
HLA-DR^+^ CD38^+^	− 0.691	0.142
T CD3^+^CD8^+^		
HLA-DR^+^	− 0.585	0.141
CD38^+^	+ 1.260	0.532
HLA-DR^+^ CD38^+^	+ 1.189	0.876

Data show correlations between basal and G2-S16 dendrimer ETP T cell activation markers on T cell CD4^+^ and CD8^+^ populations. Datas represent mean of 5 ectocervix explants performed on 4 ETP/condition. *P* = *p* value of unpaired T-test between non-treated tissue and G2-S16 dendrimer 3 mM treatment.

**Fig. 4:**
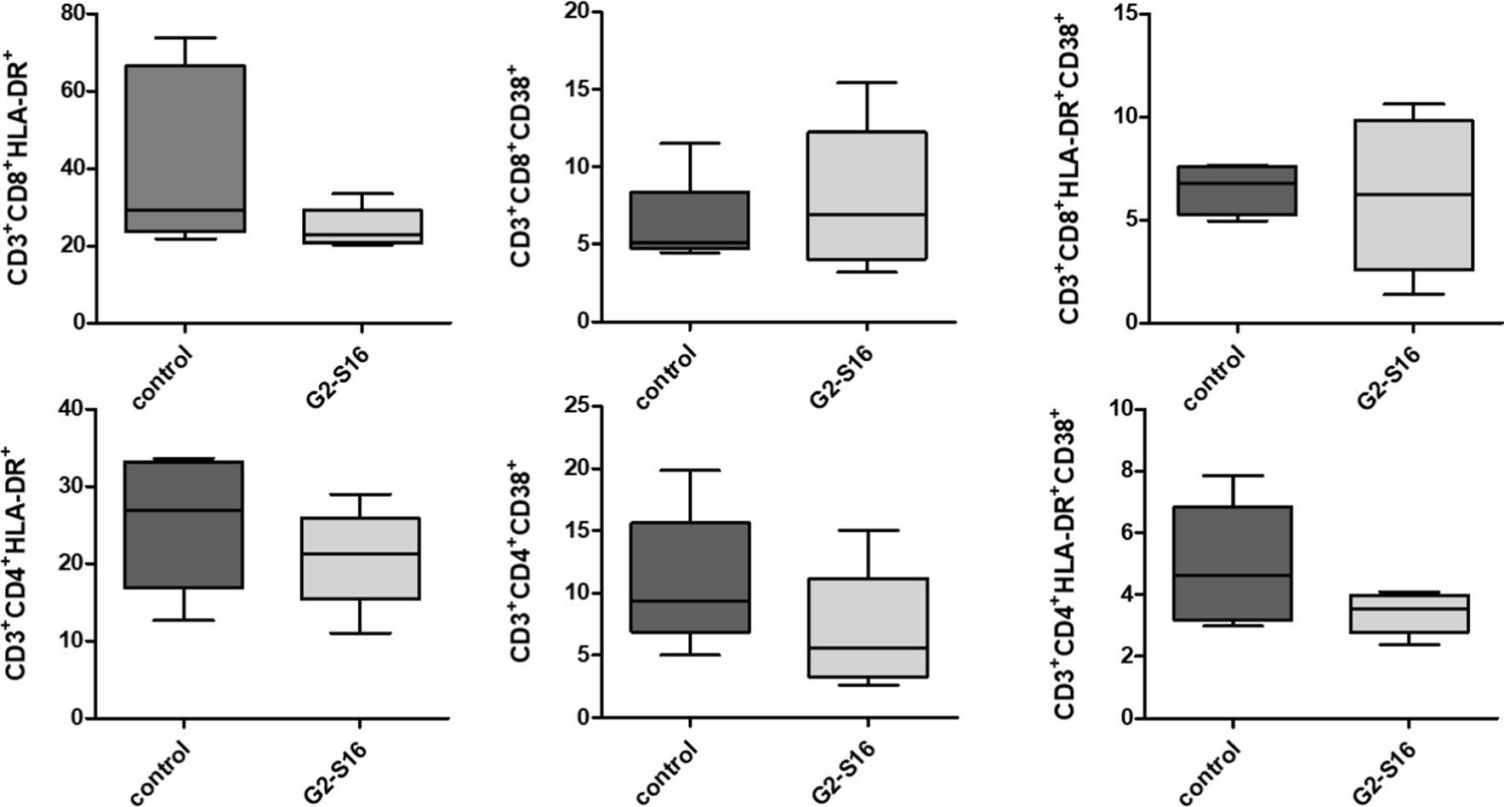
Effect of G2-S16 dendrimer treatment on T cell activation. ETPs were treated with G2-S16 dendrimer 3 mM, digested, disaggregated and analyzed by flow cytometry for T cell activation markers. Data represent effect on G2-S16 dendrimer on ETP T cell activation markers on T cell CD4 ^+^ and CD8^+^ populations. Bars represent the mean of 5 ectocervix explants ± SD

No significant differences were observed either on CD8^+^ or CD4^+^ activation, however, a slight trend could be observed on CD4^+^ population (Fig. 4, lower panels), suggesting the biosafety of G2-S16 dendrimer and diminishing possible side effects. No differences were either observed in other survival and functionality markers such as CD127 (*data not shown*).

### Histopathological analysis of G2-S16 dendrimer treatment on ETP

Vaginal explants were treated with G2-S16 dendrimer (3 mM) and histopathological damage was measured and then compared to non-treated tissues. Tissues were H/E staining and analyzed for the existence of lesions in vaginal tissue (Table 2). Damage score was assigned as described (0–4) from no lesions to a massive lesion occupying most of tissue.

**Table 2:** Vaginal explant histopathological analysis

	NT ^1^									G2-S16 (3 mM)									N9 (4.5%)				
Epithelial injury	1	1	2	2	0	2	2	2	1	2	2	0	2	1	1	2	1	2	4	4	3	4	4
Glandular cystic dilation	1	0	0	0	0	1	0	0	0	0	0	0	1	0	0	0	0	0	0	0	0	0	0
Inflammatory infiltrate	0	1	0	0	1	1	0	0	1	1	0	2	1	0	0	0	0	1	0	2	0	0	0
Hemorrhage	1	0	0	0	1	0	0	0	0	1	0	0	0	0	0	0	0	0	0	0	0	0	0
Vascular proliferation	0	2	2	0	0	1	2	2	1	1	2	1	1	0	2	1	1	0	3	4	4	3	4
Edema/fibrosis	0	0	0	0	0	0	0	0	0	0	0	0	0	0	0	0	0	0	0	0	0	0	0
Necrosis	1	2	1	0	0	1	0	2	0	1	2	1	1	1	2	1	0	1	3	4	3	3	4
Calcifications	0	0	0	0	0	0	0	0	0	0	0	0	0	0	0	0	0	0	0	0	0	0	0
Total score	4	6	5	2	2	6	4	6	3	5	6	4	6	1	5	4	2	4	10	14	10	10	12

Injury existence was evaluated in each ETP sample for epithelial injury, glandular cystic dilation, inflammatory infiltrate, hemorrhage, vascular proliferation, edema or fibrosis, necrosis and calcifications

^**1**^ 0 (no change); 1 (minimum); 2 (light); 3 (moderate); 4 (very serious). Total score summatory set the histopathological analysis score as minimum 1–6, average 7–10, moderate 10–14, and severe 14 + .

*N9 *nonoxynol-9

**Fig. 5:**
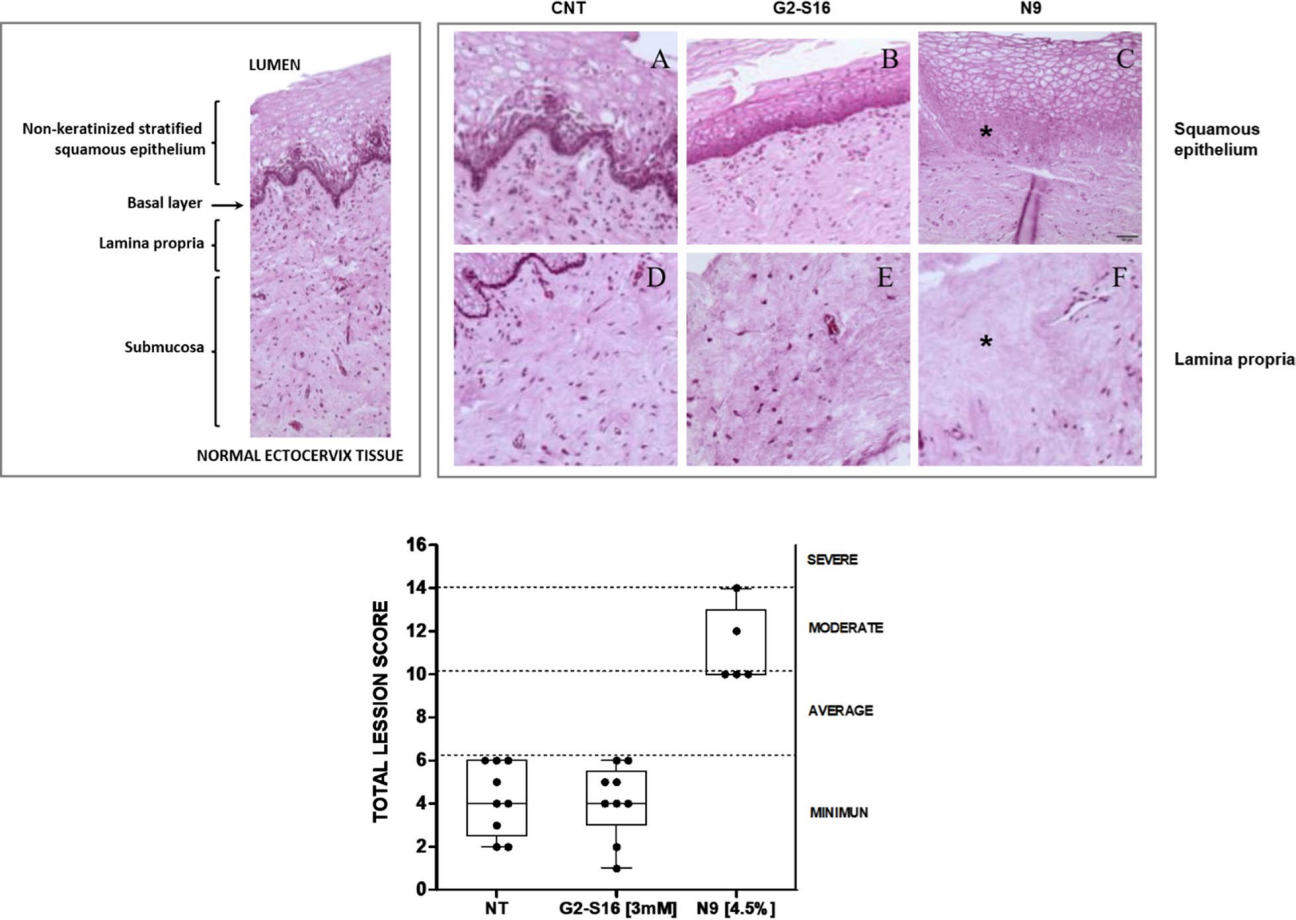
Histopathological analysis. Total accumulative score of each ETP analyzed (Lower graphic). Data represent summary of each individual damage score analyzed for ETP non-treated or G2-S16 dendrimer 3 mM treatment. N9 4.5% was used as tissue damage control. (Upper graphic) Representative images of normal vaginal ectocervix tissue (Left graphic) and Representative images of non-treated ETP (**A**, **D**) G2-S16 3 mM treatment (**B**,** E**) and N9 4.5% (**C**, **F**). Each represented point corresponds to ETP vaginal explants. *NT* non-treated; *N9* Nonoxinol-9; 4.5% w/v

All tissues analyzed the control group present scarce lesions located mainly at the epithelium level, with mucosa proliferation or detachment of superficial cells (stratum lucidum and corneal) (Fig. 5A, D). Related to G2-S16 dendrimer treatment, the mucosal epithelium appears detached from the submucosa in most analyzed tissues or a space is observed between both layers (Fig. 5B, E), probable due to tissue processing. Additionally, lesions in the analyzed tissues were similar to those observed in non-treated ETP.

Treatment with N9 (Fig. 5C, F) presented necrosis of the epithelium, especially the basal ones (star in C) and and areas of necrosis in submucous (star in F).

Finally, no statistical differences were observed in the total score between non-treated and G2-S16 dendrimer treatment (Fig. 5). This data confirms the biosafety of G2-S16 dendrimer in human vaginal explants.

## Discussion

New treatments against HIV-1 pandemic have been improved during last decades and fortunately nowadays patients are capable of controlling viral load as well as AIDS progression [26]. The 2020 program proposed 90, 90, 90 strategies which means that 90% of population living with HIV-1 would be diagnosed, 90% of those would receive the treatment and 90% of those would have suppressed viral load for the year 2020 [2]. Unfortunately, this goal has not been achieved and even more, an upturn of new infections has been detected in last years, mainly related to risky sexual practices [2].

In this sense, prophylaxis pre-exposure (PreP) was an important advance in epidemic control, since this daily treatment for HIV-1 negative people, who perpetrate risky sexual conduct, kept preventing them from the HIV-1 infection [27]. PreP was approved of by the United States in 2012 and recommended for the European Center for Disease Prevention and Control (eCDC) in 2015. However, a great portion of nations has not approved of this strategy as the use of these treatments presents several controversies [28, 29].

To develop an effective, biosafe, self-administrated, inexpensive and easy dispense topic microbicide against HIV-1 infection could fill this gap in prophylactic treatments and subserve control of new infections. The main objective of this work was to confirm the previously in vitro and in vivo obtained results with the vaginal G2-S16 dendrimer gel microbicide.

Our investigation group previously demonstrated the efficacy and biocompatibility of G2-S16 dendrimer against HIV-1 infection, the underlying mechanism of action [30], and even more, G2-S16 does not generate resistance mutations due to continued treatment [31]. In this work efficacy, biosafety and possible side effects of G2-S16 dendrimer treatment have been evaluated in the most reliable clinical model, healthy human vaginal explant. Vaginal explants obtained from healthy women mimic-real-life conditions, tissue behavior, immune response in situ, and possible irritation or inflammation effects on histopathological analysis. The fact that the tissue explants proceeded from human healthy donors poses these explants as a great model better than mice models and other in vivo assays.

Firstly, each tissue explant was assayed for a previous viability test to ensure the perfect status of the tissue for the incoming experiments. Once tissue status was confirmed, tissues were processed ETP as described (Fig. 1A).

To set the maximum non-toxic concentration for G2-S16 dendrimer in tissue explants was the first and crucial step of the study. ETP treatment with G2-S16 dendrimer located 3 mM concentration for G2-S16 dendrimer as the maximum non-toxic concentration and was determined for the incoming issues (Fig. 1B).

The effect of the dendrimer against HIV-1 infection could be masked due to tissue toxicity of the treatment, infection or co-treatment and infection. If viral infection promotes tissue damage or cellular death, infection values and subsequent inhibition could be untrue, or if the treatment with G2-S16 dendrimer generates side effects on tissue behavior, when HIV-1 infections take place, inhibition observed on data curate could be due to the cellular or tissue death and not directly to a real effect of G2-S16 dendrimer inhibition. To rule out this possibility, viability assay was performed in every experimental condition. Data demonstrated that any of the treatments proposed (G2-S16 dendrimer treatment, HIV-1 infection and co-treatment and infection G2-S16 dendrimer-HIV-1) do not affect tissue viability either at 2- or 5-days post-infection (Fig. 1C). Even more, at 5 days post-infection, co-treatment G2-S16 dendrimer-HIV-1 enhanced ETP viability compared to HIV-1 infection alone, probably due to the protective and inhibitory effect of G2-S16 dendrimer (Fig. 1C).

Next step was to confirm the inhibitory effect of G2-S16 dendrimer shown previously in vivo assays. ETP were treated with G2-S16 dendrimer and HIV-1 infected. Supernatants were titrated on TZM.bl cell line for HIV-1 inhibition and ETP were both assayed for viability to rule out the mentioned masking possibilities, and disaggregated for cytometry assays. Data obtained confirm the inhibition of G2-S16 dendrimer as well at 2 days post-infection as at 5 days post infection (Fig. 2). Viability confirmation also demonstrated a direct effect of G2-S16 dendrimer treatment due to the survival of tissues (Fig. 2).

Once the inhibition and the biosafety of G2-S16 dendrimer treatment were demonstrated, the next step was to assess the proper T cell population ratio and behavior. ETP were disaggregated and immune-stained for CD3, CD4, CD8 and aqua^®^ (positive dead staining). Data shown that HIV-1 infection does not alter the CD3^+^ CD4^+^ and CD3^+^ CD8^+^ ratios (Fig. 3A, lower panels). However, when tissues were treated with G2-S16 dendrimer and HIV-1 infected, CD3^+^CD4^+^ population disappeared. Surprisingly, when CD4^+^ population was defined as CD3^+^ CD8^−^, the populations remain unalterable and do not present alterations compared to HIV-1 infection. This behavior is due to the own action of the dendrimer. G2-S16 dendrimer joins CD4 receptor and destabilizes CD4-Gp120 interactions, blocking this first union of the virus with the membrane receptors of the host cell, avoiding the membrane fusions and the subsequent infection, as we have previously described by molecular modeling as well as in in vitro models [30]. This non-expected result strongly confirms that G2-S16 dendrimer described mechanism of action against HIV-1 infection in real-life human conditions.

To reconfirm and complete the biosafety data obtained for G2-S16 dendrimer treatment and to rule out possible side effects once the dendrimer is raised to clinical trials, vaginal explants were assessed for immune activation. Tissue explants were treated with G2-S16 dendrimer and compared to non-treated tissues. ETP were disaggregated and immune-stained for CD3, CD4 and CD8; HLA-DR and CD38 as activation markers and aqua^®^ as viability control. As shown in Fig. 4 and Table 1, no statistical differences were observed between non-treated ETP and G2-S16 dendrimer treatment. These data confirm that G2-S16 dendrimer treatment does not generate an immune activation either on CD4^+^ or CD8^+^ population, establishing G2-S16 dendrimer as a safe treatment. Even more, on CD4^+^ population a mild reduction on activation levels as well in HLA-DR^+^, CD38^+^ as in double positive HLA-DR^+^ CD38^+^ staining was observed (Table 1). This is very interesting data since several accurate treatments generate an hyperactivation of the immune system, leading into an immune exhaustion and the subsequent precocious immune system senescence.

Finally, we attempt to support and reconfirm all collected data related to the biosafety and effectiveness of G2-S16 dendrimer treatment with histopathological assays. ETP were H/E stained and assayed for tissue damage. Vaginal explants were analyzed for epithelial injury, glandular cystic dilation, inflammatory infiltrate, hemorrhage, vascular proliferation, edema or fibrosis, necrosis and calcifications. Damage scores were assigned to each parameter from no changes (0) to a generalized damage (4). All individual scores were added for each tissue and final accumulative scores were assigned to each tissue from minimum (1–6), to severe (14 +). Each individual and cumulative values for each sample were recorded in Table 2.

Histopathological analysis demonstrated that G2-S16 dendrimer treatment does not affect the epithelial injury, glandular cystic dilation, inflammatory infiltrate, hemorrhage, vascular proliferation, edema or fibrosis, necrosis and calcifications. Total scores recording for G2-S16 dendrimer locates tissue damage due to G2-S16 dendrimer treatment as minimum (Fig. 5). Tissue damage and toxicity control N9 indicates that tissues were susceptible to going through alterations in analyzed parameters and the severe tissue damage was generated (Table 2 and Fig. 5).

As shown in Fig. 5 AD, tissue damage was mainly located in the epithelial layer, where the majority of tissues presented alterations and were detached from the submucosa, even in non-treated tissues. This fact could take place due to the tissue extraction process as well as to the ETP preparation. However, as demonstrated in Fig. 5 A–D tissues present a healthy appearance, they neither infiltrate nor show vascular alternations, nor any necrosis significant areas.

## Conclusion

Summing up, all the presented results in this study confirm the biosafety and efficacy of G2-S16 dendrimer in human healthy vaginal explants. All these data together with previously obtained on this dendrimer, strongly support the need of enrolling G2-S16 dendrimer vaginal prophylactic treatment in a clinical trial.

This first clinical step was enormously valuable to invite to contemplate possible positive results in further clinical trials. G2-S16 dendrimer treatment has few side effects and it is notably well accepted due to its characteristics in the topical microbicide.

## Material and methods

### Dendrimer, cells and viral isolates

**Fig. 6:**
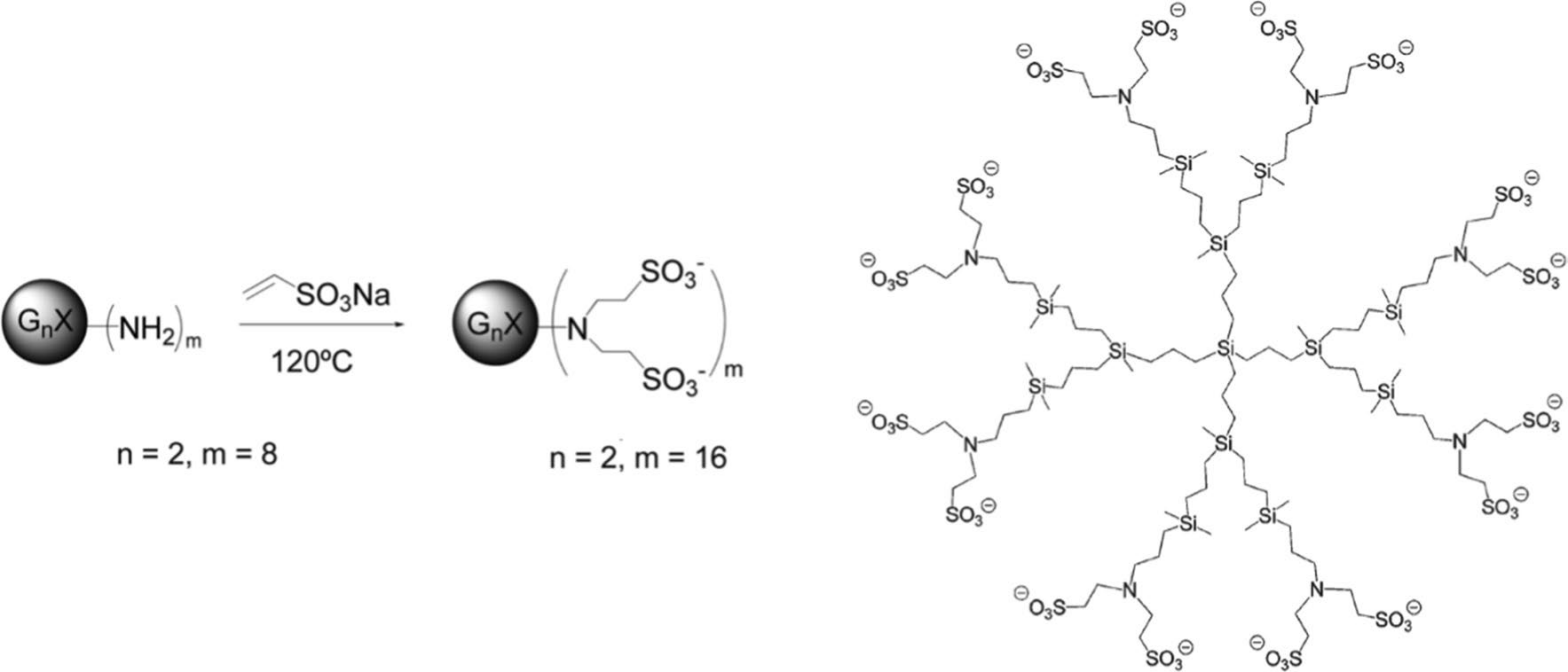
G2-S16 polyanionic carbosilane dendrimer. Schematic representation of G2-S16 dendrimer and synthesis

G2-S16 dendrimer was characterized and synthesized as previously described [32] (C_112_H_244_N_8_Na_16_O_48_S_16_Si_13_; Mw: 3717.15 g/mol). General characteristic of G2-S16 dendrimer were described in Fig. 6 and Table 3. Lyophilized dendrimer was dissolved in distilled water to mM concentration for further assays.

**Table 3:** Previously described and characterized properties of G2-S16 dendrimer

G2–S16 phisicochemical properties	
General characteristic	
Molecular formula	C_112_H_244_N_8_Na_16_O_48_S_16_Si_13_
Molecular weigh	3717.15 g/mol
Structure	Anionic carbosilane dendrimer
Generation	G2
Core	Silica
Functional groups	SO_3_^−^
Number of peripheral groups	16
Structural parameters	
R [nm]	1.767 ± 0.103
Rg [nm]	0.932 ± 0.020
SASA [nm^2^]	33.63 ± 1.45
SESA [nm^2^]	24.76 ± 0.33
SEV [nm^3^]	3.330 ± 0.073
SPHER	0.938 ± 0.024
Aqueous solutions parameters	
Z potential	− 74.0 mV
Diffusion coefficients (*D*, 10^−11^ m^2^/s)	4.99
Hydrodynamic radii (r_H_, nm)	4.29

*R* Molecular radius, *Rg* radius of gyration, *SASA* solvent accessible surface area, *SESA* solvent-excluded surface area, *SEV* solvent-excluded volume, *SPHER* sphericity

TZM.bl cells were obtained from NIH AIDS Research and Reference Reagent Program (Germantown, MD, USA), and cultured in Dulbecco’s Modified Eagle’s Medium (DMEM) (Biochrom GmbH, Berlin, Germany) with 5% heat-inactivated fetal bovine serum (FBS) (Biochrom GmbH, Berlin, Germany), and a cocktail of antibiotics (125 mg/mL ampicillin, 125 mg/mL cloxacillin and 40 mg/mL gentamicin (Normon, Madrid, Spain).

Viral stocks of the THRO transmitter founders (T/F) virus was obtained after transfection of 293 T cells (ATCC, CRL^®^ -216TM; Manassas, VA, USA) with pTHRO.c/2626 plasmids (NIH AIDS Research and Reference Reagent Program). Viral stocks were clarified by centrifugation prior to evaluation of the viral TDCID50 titer using a HIV p24^gag^ ELISA (Innotest^®^ HIV, antigen mAb, Innogenetic, Ghent, Belgium).

### Ethics

Study was performed in accordance with local ethical regulations, following approval by the local ethical committee (CEIm Hospital Viamed Santa Angela de la Cruz, Sevilla.; Project code: VAG.EX-01). After project acceptance by the CEIm, all study participants provided the written informed consent.

### Participants inclusion and tissue recollection

Human vaginal tissue explants were obtained from healthy women between 18–50 years old who undergo hysterectomy for prolapse or uterine myoma at the Viamed Santa Angela de la Cruz Hospital, Seville. Hysterectomy surgeries performed for benign pathologies. Women who at the time of hysterectomy are over 50 years old, pregnant, have vaginal or extravaginal neoplasia, active systemic infection or have received corticosteroid treatment in the last 6 months, have an intrauterine device implanted, use vaginal ovules, hormonal contraceptives or have no menstruation will be excluded from the study. A total of 25 vaginal tissue explants were obtained; Three of them were excluded since unsuitable tissues viability. Remaining tissues were processed as follows.

Tissue is transported by ECM transport medium (RPMI Biochrom GmbH, Berlin, Germany + 15% FBS + 1% Amino Acids (Gibco, Amarillo, Texas, USA) + 1% MEM Pyruvate (Gibco, Gibco, Amarillo, Texas, USA) + 2.5 µg/mL Amphotericin (Gilead, Madrid, Spain) and 40 µg/mL Gentamicin (Normon, Madrid, Spain)) and processed in the laboratory no later than 24 h after the operation. Fat layer is removed from the muscle tissue of the cervix, leaving the area of the ecto and endocervix as clean as possible. The ectocervix is separated from the endocervix, since the endoocervix continues producing mucus in culture and could interfere with future results. The ectocervix is processed into ectocervix tissue portions (ETP) of approximately 2mm × 2mm, transferred to a clean p100 plate and washed in fresh ECM (Fig. 1A).

### Viability and cytotoxicity assay

Once tissue was processed, ETP were transferred to a p24 well plate and treated with G2-S16 dendrimer in a concentration range (0.5–4 mM) for 48 h or 5 days. After treatment, 500 µL of MTT(3-(4-5-dimethylthiazol-2-yl)-2,5-diphenyltetrazolium bromide) (Sigma, St Louis, MO, USA) (2.5 mg/mL dissolved in optimum) were added. After 3 h, ETP are transferred to 500 µL of isopropanol to dissolve the MTT crystals and ETP were incubated at RT for 48 h. Absorbance is read at 490 nm on a spectrophotometer Synergy 4 plate reader (BioTek, Winooski, VT, USA).

### HIV-1 infection

Viral infection and prophylactic activity were evaluated. Four ETP were transferred for each eppendorf in 500 µL of ECM, and infected with viral isolate THRO (founder) with 7500 TCID50 for 3 h a thermoblock at 37ºC and 400 rpm., or 1 h of pretreatment with 3 mM G2-S16 dendrimer and subsequently infected 3 h with viral isolate THRO (7500 TCID50) in a thermoblock at 37 ºC and 400 rpm.

After infection, a 1 × 1 cm gelatin hemostatic pad (Topiston®) is placed in a p12, and 1 mL of ECM is added to hydrate the pad during the 3 h of infection. After infection, ETP from each condition were transferred to a p12 well plate, and placed on the hydrated pad. The infection volume (500 µL of each eppendorf) is recovered and added to each well. Sections are incubated for 72 h. Supernatants were collected for titration on TZM.bl cell line, and ETP were assayed for viability as mentioned above.

TZM.bl cells previously seeded on DMEM 5% FBS were treated with 100 µL of supernatants and incubated 2 h. Plates were washed (PBS) and fresh DMEM 5% FBS medium was replaced. After 48 h incubation, TZM.bl cells were lysed and luciferase activity was measured according to manufacture instruction. Luminescence was recorded at 135/200 nm with BioTek Synergy™ 4 Hybrid Microplate Reader.

### Tissue digestion

Single-cell suspension from tissue disaggregation was obtained after ETP treatments as mentioned above. Once ETP were treated, they were transferred to an eppendorf with 500 µL of ECM + 500 µL of collagenase IV (10 mg/mL) (Gibco, Amarillo, Texas, USA), and incubated 30 min in a thermoblock at 37 °C and 400 rpm. 500 µL of supernatant were filtered and transferred to a new eppendorf. Tissues were mechanically broken down into the remaining 500 µL with the plunger of a 1 mL syringe. Collagenase is neutralized with 500 µL of PBS and the remaining volume is filtered to the new eppendorf. Filtrated volumes were centrifuged 5 min at 1500 rpm.

### Flow cytometry

The filtered supernatants were resuspended in PBS + 2% FBS and immunostained for viability with Aqua^®^ (Invitrogen, Waltham, Massachusetts, USA) and incubated 5-10 min at 4 º. After labeling with Aqua, mix was prepared with the rest of the antibodies. The monoclonal antibodies directed against human leukocyte surface markers used were as follows: CD57-fluorescein isothiocyanate, CD28-phycoerythrin, (Beckman Coulter Company, Immunotech, France), CD4-allophycocyanin-cyanine 5, CD38-allophycocyanin-cyanine 7 (eBioscience, San Diego, CA), human leukocyte antigen-D related (HLADR)-allophycocyanin, CD8-Pacific Blue (BioLegend, San Diego, CA) and CD3-Pacific Orange (Invitrogen, Carlsbad, CA). Cellular activation in CD4 and CD8 T cells was characterized by HLA-DR^+^ and CD38^+^ coexpression. PBS + 2% FBS was used to complete a final volume of 100 µL per condition. 100 µL of the mix were added on the label with Aqua and incubated 45 min at 4º. After incubation, 1 mL of PBS + 2% FBS was added and centrifuged 5 min at 1500 rpm. Supernatants were removed and resuspended in 200 µL + 200 µL of 4% PFA.

Samples were analyzed in a Navios EX flow cytometer (Beckman Coulter, Brea, CA, USA) and data obtained were analyzed using Kaluza Flow Analysis Software (Beckman Coulter, Brea, CA).

### Histopathology analysis of ETP

Hematoxylin–eosin (H/E) staining for histopathological damage were assayed on ETP sections treated with G2-S16 dendrimer. This assay allows to determine the presence of histological lesions in the vaginas. ETPs were embedded in paraffin by passing through alcohols of increasing degree, two xylene baths and one paraffin bath, and ETP were placed in a paraffin mold. They were subsequently processed using a microtome and staining. For the dewaxing of the samples, two xylene baths (10 min) and three alcohol baths were used in decreasing direction (100%, 90% and 70%) (5 min). ETPs were stained with hematoxylin (5 min) and eosin (5 min). During the dehydration process after eosin staining, alcohols in increasing solution (70%, 96% and 100%) and xylene were used. Samples were finally mounted with DPX and analyzed under light microscopy.

Each ETP sample was analyzed for the existence of lesions in the epithelium of the vagina, the inflammatory infiltrate, vascular congestion. The assigned values (score) was assigned as follows: lesions were 0 (no changes): when no lesions were observed or the observed changes were within normality; 1 (minimum): when the changes were scarce but exceeded those considered normal; 2 (slight): the lesions were identifiable but with moderate severity; 3 (moderate): significant injuries, but can still increase in severity; 4 (very serious): the lesions are very serious and occupy most of the tissue analyzed.

### Statistical analysis

Statistical study, including mean, standard deviation and significant differences were analyzed using GraphPad Prism v5.0 software (GraphPad, San Diego, CA, USA). Data shown and analysis carried out in each assay, as well as *n* size are properly described in its corresponding section. The significance, solved at *p* value ≥  0.05, was determined by using t-test of unpaired values and a non-parametric test.
